# Hybrid Convergent ablation for atrial fibrillation: A systematic review and meta-analysis

**DOI:** 10.1016/j.hroo.2022.05.006

**Published:** 2022-05-16

**Authors:** Suvash Shrestha, Kristen M. Plasseraud, Kevin Makati, Nitesh Sood, Ammar M. Killu, Tahmeed Contractor, Syed Ahsan, David B. De Lurgio, Christian C. Shults, Zayd A. Eldadah, Andrea M. Russo, Bradley Knight, Yisachar Jesse Greenberg, Felix Yang

**Affiliations:** ∗ Maimonides Medical Center, Brooklyn, New York; † AtriCure, Inc, Mason, Ohio; ‡ Tampa Cardiac Specialists, Tampa, Florida; § Southcoast Health System, Fall River, Massachusetts; ‖ Mayo Clinic, Rochester, Minnesota; ¶ Loma Linda University Medical Center, Loma Linda, California; # St. Bartholomew’s Hospital, London, United Kingdom; ∗∗ Emory St. Joseph’s Hospital, Atlanta, Georgia; †† Medstar Washington Hospital Center, Washington, District of Columbia; ‡‡ Cooper Medical School of Rowan University, Camden, New Jersey; §§ Northwestern University, Chicago, Illinois

**Keywords:** Atrial fibrillation, Hybrid ablation, Electrophysiology, Surgical ablation, Meta-analysis

## Abstract

**Background:**

Hybrid Convergent ablation for atrial fibrillation (AF) combines minimally invasive surgical (epicardial) and catheter (endocardial) ablation. The procedural goal is to achieve more extensive, enduring ablation of AF substrate around the pulmonary veins, posterior wall, and vestibule of the posterior wall left atrium.

**Objective:**

To perform a systematic review and meta-analysis on safety and effectiveness of contemporary Hybrid Convergent procedures.

**Methods:**

PubMed, Embase, and manual searches identified primary research articles on Hybrid Convergent. Inclusion criteria focused on contemporary practices (epicardial ablation device and lesions). Clinical outcomes at 1 year or later follow-up, patient population, procedural details, and major adverse events (MAE) were recorded.

**Results:**

Of 249 records, 6 studies (5 observational, 1 randomized controlled trial) including 551 patients were included. Endocardial energy sources included radiofrequency and cryoballoon. Hybrid Convergent ablation was mostly performed in patients with drug-refractory persistent and longstanding persistent AF. Mean preprocedural AF duration ranged between 2 and 5.1 years. Most patients (∼92%) underwent Hybrid Convergent in a single hospitalization. At 1 year follow-up or later, 69% (95% confidence interval [CI]: 61%–78%, n = 523) were free from atrial arrhythmias and 50% (95% CI: 42%–58%, n = 343) were free from atrial arrhythmias off antiarrhythmic drugs. Thirty-day MAE rate was 6% (95% CI: 3%–8%, n = 551).

**Conclusion:**

Hybrid Convergent ablation is an effective ablation strategy for persistent and longstanding persistent AF. Contemporary procedural approaches and published strategies aim to mitigate complications reported in early experience and address delayed inflammatory effusions.

**Key Findings**

▪ In patients who had Hybrid Convergent ablation with contemporary methods, 69% were free from atrial arrhythmias and 50% were free from atrial arrhythmias off antiarrhythmic drugs at 1 year or longer after the procedure.
▪ Three studies showed that a significantly greater proportion of patients experienced low residual atrial fibrillation (AF) burden through 12 or more months after Hybrid Convergent.
▪ We found with Hybrid Convergent ablation that major adverse events (MAEs) within 30 days occurred at a pooled rate of 6%, which was numerically higher than those undergoing endocardial catheter ablation alone. However, across studies, individual MAEs occurred at rates in line with expected estimates for AF-related ablation.

## Introduction

Cardiac ablation to achieve electrical isolation of the pulmonary veins (PV)^1^ and the posterior left atrial (LA) wall^2^ has become a cornerstone of atrial fibrillation (AF) treatment. Whereas success rates in catheter-based ablation of paroxysmal AF are generally reported to be over 70% with intermediate-term follow-up,^3^, ^4^, ^5^, ^6^, ^7^ success rates in patients with persistent AF (PersAF) are markedly lower, presenting a challenge for treatment.^8^

Surgical epicardial and electrophysiological endocardial ablation are 2 approaches for AF ablation. A surgical epicardial approach allows for the creation of more efficient ablation lesions that are larger and potentially more transmural, while limiting the risk of collateral injury. Additionally, a surgical approach enables left atrial appendage (LAA) exclusion and isolation. However, limitations to a surgical approach include lack of substrate mapping and an inability to reliably confirm isolation across the veins and ablation lines. An endocardial, catheter-based approach enables ablation of triggers for AF that may otherwise go untreated, as well as confirmation of transmurality and completion of the intended lesion set. Therefore, a hybrid approach that harnesses both epicardial and endocardial ablation strategies may provide a more effective, enduring therapy for AF than the singular approach.^9^ With a hybrid approach, the epicardial portion can be performed thoracoscopically or endoscopically through a subxiphoid incision. The Hybrid Convergent procedure, which uses a subxiphoid incision, was the ablation strategy used in the prospective, randomized CONVERGE clinical trial.^10^^,^^11^

Given emerging evidence on the safety and effectiveness of Hybrid Convergent ablation and recent FDA approval of the EPi-Sense Guided Coagulation System (AtriCure, Inc, Mason, OH), we conducted a systematic review of contemporary published studies on outcomes of this procedure in PersAF and longstanding persistent AF (LSPAF).

## Methods

We followed the PRISMA statement for reporting systematic review and meta-analysis in this study.

### Data sources and search criteria

The following keywords were used for the PubMed database search: atrial fibrillation AND (“hybrid ablation” OR “convergent ablation” OR “hybrid procedure” OR “convergent procedure” OR “epicardial-endocardial ablation” OR “surgical electrophysiological approach”). For the Embase search, the keyword search string was (convergent AND ablation AND atrial AND fibrillation). We included studies involving only the convergent procedure, which used a transdiaphragmatic or subxiphoid approach to access the left posterior atrial wall for the epicardial ablation portion followed by endocardial catheter ablation. Studies using a thoracoscopic approach for epicardial ablation in hybrid procedures and procedures involving concomitant cardiac surgeries such as valve surgery or coronary artery bypass graft were excluded. We also reviewed the reference lists of the shortlisted articles published before May 2021 to identify other potentially relevant articles. The analysis was further restricted to studies that used the current-generation unipolar radiofrequency (RF) device (EPi-Sense; AtriCure, Inc, Mason, OH) to create parallel linear lesions across the posterior wall of the left atrium during the epicardial portion of the procedure. Studies using previous-generation devices (eg, Visitrax, Numeris) and/or a box lesion set (eg, extra-maze lesion set) were excluded. The rationale for this restriction was to reflect contemporary practice aligned with the CONVERGE clinical trial.^10^^,^^11^

### Study selection

Two authors (SS, KP) individually and independently conducted the initial screening of the list examining the titles and abstracts and removed other articles except original research articles. We had no restrictions for publication date. We included only articles published in English. Case reports and series with fewer than 10 patients, editorials, conference abstracts, and reviews were excluded. Full-text articles were retrieved for those shortlisted and 2 authors (SS, KP) reviewed them in detail to ensure they met the inclusion criteria and collected relevant data. Duplicates were removed. In case of studies from the same authors, only the most recent article was included.

### Data extraction and analysis

We recorded data on the following variables: patient characteristics, procedural details, concurrent antiarrhythmic drug (AAD) use, documented major adverse events (MAE), rhythm monitoring type and frequency, and freedom from atrial arrhythmias at 1 year or later post procedure. MAEs were defined as those specified in the CONVERGE protocol, derived from the 2017 HRS consensus statement, which included cardiac tamponade/perforation, severe PV stenosis, excessive bleeding (requiring reoperation or transfusion with ≥2 units of packed red blood cells), myocardial infarction, stroke, transient ischemic attack, atrioesophageal fistula (AEF), phrenic nerve injury, and death.^12^ Meta-analysis was performed using a random-effects model with a restricted maximum likelihood estimator and forest plots. Heterogeneity was tested using Cochran’s Q test. Meta-analysis was performed in R version 3.6.3 with the metafor package (version 2.4-0). Estimation is based on restricted maximum likelihood via a random-effects model to allow for potential treatment heterogeneity. Raw proportions were analyzed for ease of interpretation.

### Quality and risk-of-bias assessment

Two authors independently assessed the quality and risk of bias of the included articles using the Cochrane Risk-of-Bias Tool for Randomized Trials 2.0 for randomized studies^13^ and the Newcastle Ottawa scale for nonrandomized cohort studies.^14^ A score of 6–9 has been suggested to indicate good study quality.^15^ Publication bias was evaluated with funnel plots.

## Results

**Figure 1:**
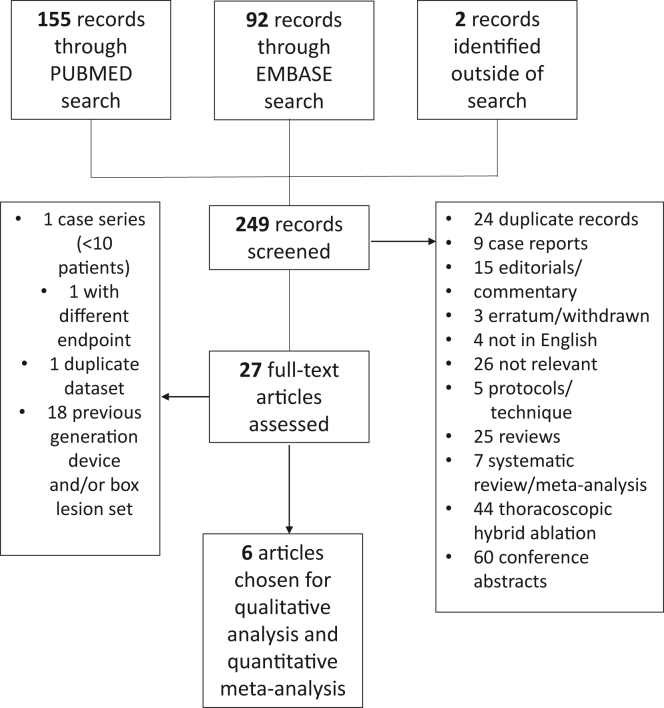
Systematic search and article selection workflow.

Initial database searches yielded 247 results (155 with PubMed and 92 with Embase), with 2 additional results from manual searching (Figure 1).^16^^,^^17^ After screening and review, 6 studies were included with a total of 551 patients.^10^^,^^18^, ^19^, ^20^, ^21^, ^22^ Five were observational studies—either prospective or retrospective—and 1 was the prospective, multicenter randomized controlled trial CONVERGE.^10^^,^^11^ Risk of bias assessments are shown in the Supplemental Data (Supplemental Tables 1 and 2).

Patient characteristics are shown in Table 1. Seventy-three percent (399/548) were male. Mean ages ranged from 61 to 69 years across studies. Mean baseline ejection fraction was ≥50% in most studies. Most (96%, 511/532) patients with documented preoperative AF classification who received Hybrid Convergent ablation had symptomatic PersAF or LSPAF. Where reported, most patients had failed at least 1 AAD. Three studies specified inclusion of some patients who had undergone prior catheter ablation for PV isolation (PVI).^19^, ^20^, ^21^ In those 3 studies, 37% (71/192) had prior catheter ablation. CONVERGE only included patients for whom hybrid convergent ablation was a *de novo* procedure,^10^ and Makati and colleagues^22^ also included some *de novo* procedures. The LA size was enlarged, with a mean size measuring greater than or equal to 4.3 cm in all studies where LA size was reported. Mean preprocedure AF duration ranged from 2 to 5.1 years. In CONVERGE trial^10^ and the study by Maclean and colleagues,^21^ the duration of PersAF was 4.4 years and 3 years, respectively. Likewise, 48% of the patients in the study by Gulkarov and colleagues^18^ and 60% of the patients in Makati and colleagues’ study^22^ had LSPAF, suggesting the longer duration of persistent AF in the study population.

**Table 1:** Demographics of the patient populations included in the Hybrid Convergent ablation studies

Study	Study population				Male, n (%)	Duration of AF (y), mean ± SD or median (IQR)	LA size (cm), mean ± SD	Prior PVI, n (%)	Age (y), mean ± SD	EF (%)	Number of failed AADs, mean ± SD
	Total, N	Paroxysmal AF, n (%)	Persistent AF, n (%)	LSPAF, n (%)							
DeLurgio et al 2020^10^	102	0 (0)	64 (63)	38 (37)	80 (78)	4.4 ± 4.8	4.4 ± 0.6	0 (0)	63.7 ± 9.6	55.3 ± 7.8	1.3 ± 0.57
Makati et al 2020^22^	226†	3 (1.5)	79 (38.1)	125 (60.4)	155 (70)	5.08 ± 6.20	NR	NR	65.1 ± 9.5	NR	NR
Maclean et al 2020^21^	43	0 (0)	0 (0)	43 (100)	32 (74)	3	4.74 ± 0.63	15 (34.8)	68.6 ± 7.7	50	NR
Gulkarov et al 2019^18^	31	0 (0)	16 (52)	15 (48)	21 (68)	2 (0.8–4.0)	4.3	NR	62	50	≥1
Larson et al 2020^20^	113	14 (12)	99 (88)		85 (75)	5.1 ± 4.6	4.8 ± 0.7	50 (44)	63 ± 8.7	50.0 ± 11.0‡ 49.0 ± 13.2§	NR
Tonks et al 2019^19^	36	4 (11)	32 (89)		26 (72)	NR	<4.0: (n = 15; 41.7%) >4.0: (n = 21; 58.3%)	6 (16.7)	61	≥50: (n = 26; 73%) 31–49: (n = 6; 16.7%) <30 (n = 4; 11%)	≥1: 100% 2: 27.8% 3: 2.8%

AAD = antiarrhythmic drug; AF = atrial fibrillation; EF = ejection fraction; IQR = interquartile range; LA = left atrium; LSPAF = longstanding persistent atrial fibrillation; NR = not reported; PVI = pulmonary vein isolation; SD = standard deviation.

† Of total 226 patients, 207 had known AF type.

‡ Group with continuous monitoring.

§ Group without continuous monitoring.

Procedural characteristics are shown in Table 2. In 5 of the 6 studies, epicardial and endocardial procedures were predominantly performed in a same-day setting, while Maclean and colleagues^21^ separated the epicardial and endocardial procedures by approximately 6 weeks. RF energy was exclusively used for endocardial ablation in 56% (306/551) of patients and cryoballoon (with or without RF) was used in 44% of patients. While most cases had a transdiaphragmatic surgical approach (67%, 370/551), most authors noted a recent shift to subxiphoid pericardial access, which was used in 33% (181/551) of cases. Length of stay was not systematically reported, but Gulkarov and colleagues^18^ reported median hospital stay of 6 days (interquartile range 5–8 days). The authors noted this may have been impacted by warfarin use, rather than procedural complications.^18^

**Table 2:** Procedural characteristics

Study	Number of sessions	Endocardial energy source	Surgical approach	Endocardial lesions
DeLurgio et al 2020^10^	Single setting	RF	TD: 66% SubX: 34%	PVI: 100% PV touch-up/common PV ablation: 38% CTI: 96% MTI: 2% Linear/focal touch-up to address gaps around PV reflections: 13%
Makati et al 2020^22^	Single setting	Cryo	TD: 71% SubX: 29%	CTI (with RF): 100%
Maclean et al 2020^21^	Staged by ∼6 weeks	RF	TD: 93% SubX: 7%	PVI: 100% CTI: 67.4% CFAE: 55.8% Roof: 41.9% MTI: 13.9% Other: 30.2%
Gulkarov et al 2019^18^	Single setting	RF	TD: 100%	PVI: 100% CFAE: 2% CTI: 61% MTI, anterior LA line, LA roof line: 52%
Larson et al 2020^20^	Mostly single setting	RF: 83% Cryo: 12% RF/Cryo: 4%	TD: 54% SubX: 46%	NR
Tonks et al 2019^19^	Single setting: 89% Staged by 1 day: 11%	RF	TD: 31% SubX: 69% LAA exclusion: 36%	PVI: 100% Roof line: 100% RA flutter line: 50% LA flutter line: 14% RA and LA flutter line: 5%

CFAE = complex fractionated atrial electrograms; Cryo = cryoballoon; CTI = cavotriscuspid isthmus; LA = left atrial; LAA = left atrial appendage; NR = not reported; PV = pulmonary vein; PVI = pulmonary vein isolation; RA = right atrial; RF = radiofrequency; SubX = subxiphoid; TD = transdiaphragmatic.

All studies included a blanking period of 3 months. Follow-up rhythm monitoring types and frequency are shown in Supplemental Table 3. In most studies, follow-up arrhythmia assessment was performed at 3, 6, and 12 months and as clinically indicated. Larson and colleagues^20^ used event monitors at 6 and 12 months for patients (19%) without continuous monitoring devices but also reviewed in-hospital and office visit electrocardiograms.^20^ Three studies included a substantial proportion of patients (64%, 221/345) who had continuous monitoring data available through use of implantable devices.^18^^,^^20^^,^^22^ Atrial arrhythmia recurrence was defined as ≥30 seconds of AF/atrial flutter/atrial tachycardia (AT) after a 3-month blanking period in most studies. One study reported freedom from AF recurrence as ≥30 seconds through the 12-month time point. The minimal limit of detection on implantable loop recorders was 2 minutes.

**Figure 2:**
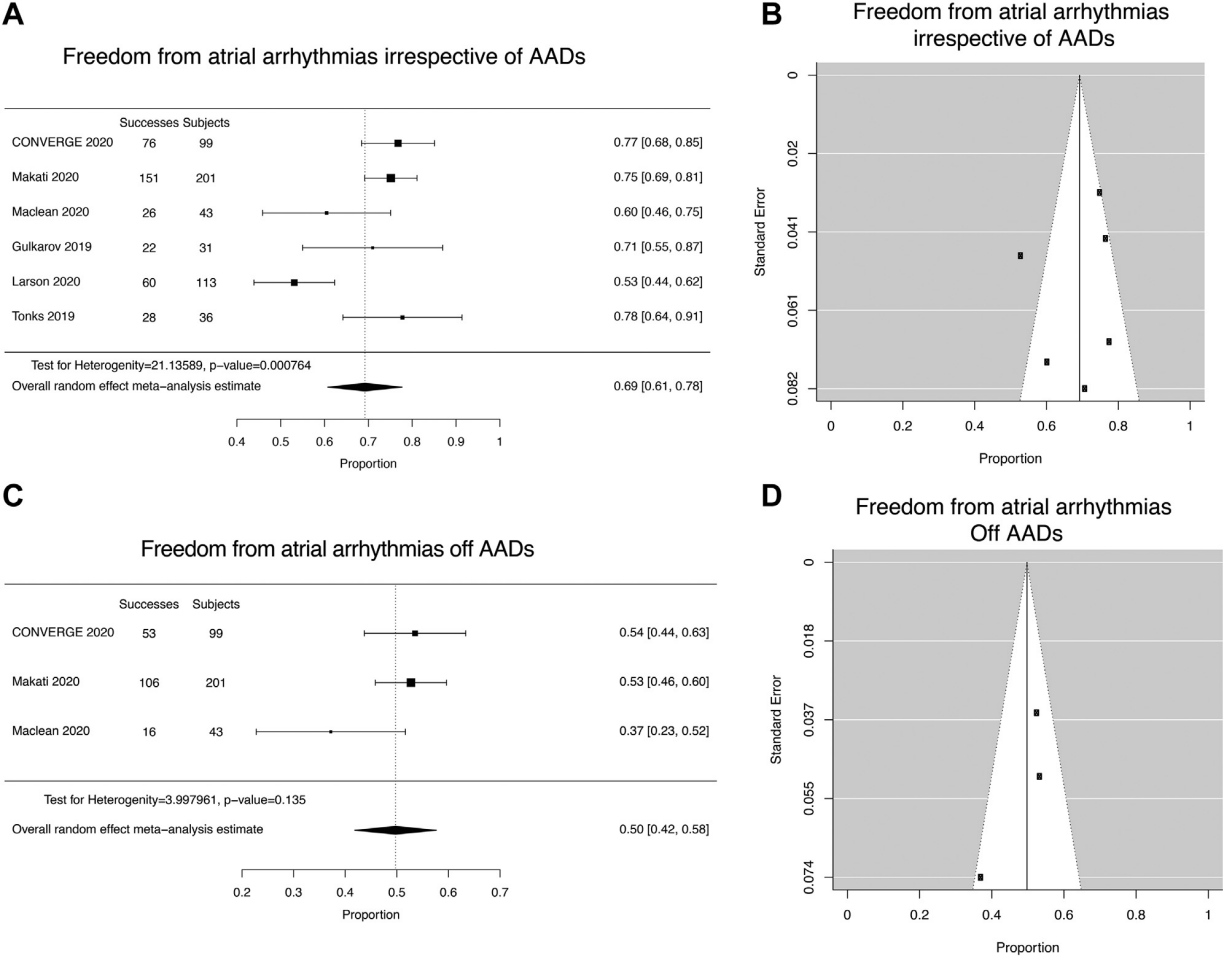
Meta-analysis of Hybrid Convergent effectiveness outcomes. **A:** Forest plot of freedom from atrial arrhythmias irrespective of antiarrhythmic drugs (AADs). **B:** Funnel plot of freedom from atrial arrhythmias irrespective of AADs. **C:** Forest plot of freedom from atrial arrhythmias off AADs. **D:** Funnel plot of freedom from atrial arrhythmias off AADs.

The effectiveness of the Hybrid Convergent procedure to eliminate recurrence from AF and/or atrial arrhythmias is shown in Supplemental Table 3 and Figure 2. In comparison with CONVERGE, where 77% of patients were free from arrhythmias irrespective of AADs and 54% free from arrhythmias off AADs, the other 5 studies ranged from 53% to 78% for freedom from arrhythmias irrespective of AADs and 2 studies ranged from 37% to 53% for freedom from arrhythmias off AADs. Meta-analysis (random-effects model) of the 6 studies found freedom from atrial arrhythmias with or without AADs at 1 year or later to be 69% (95% confidence interval [CI]: 61%–78%, n = 523; Figure 2A). The corresponding funnel plot is shown in Figure 2B. By random-effects model, 50% (95% CI: 42%–58%, n = 343) of patients in 3 studies were off AADs (Figure 2C and 2D). Heterogeneity was detected for freedom from atrial arrhythmias irrespective of AADs (Q [df = 5] = 21.1359, *P* = .0008) but not freedom from AF off AADs (Q [df = 2] = 3.9980, *P* = .1355).

**Figure 3:**
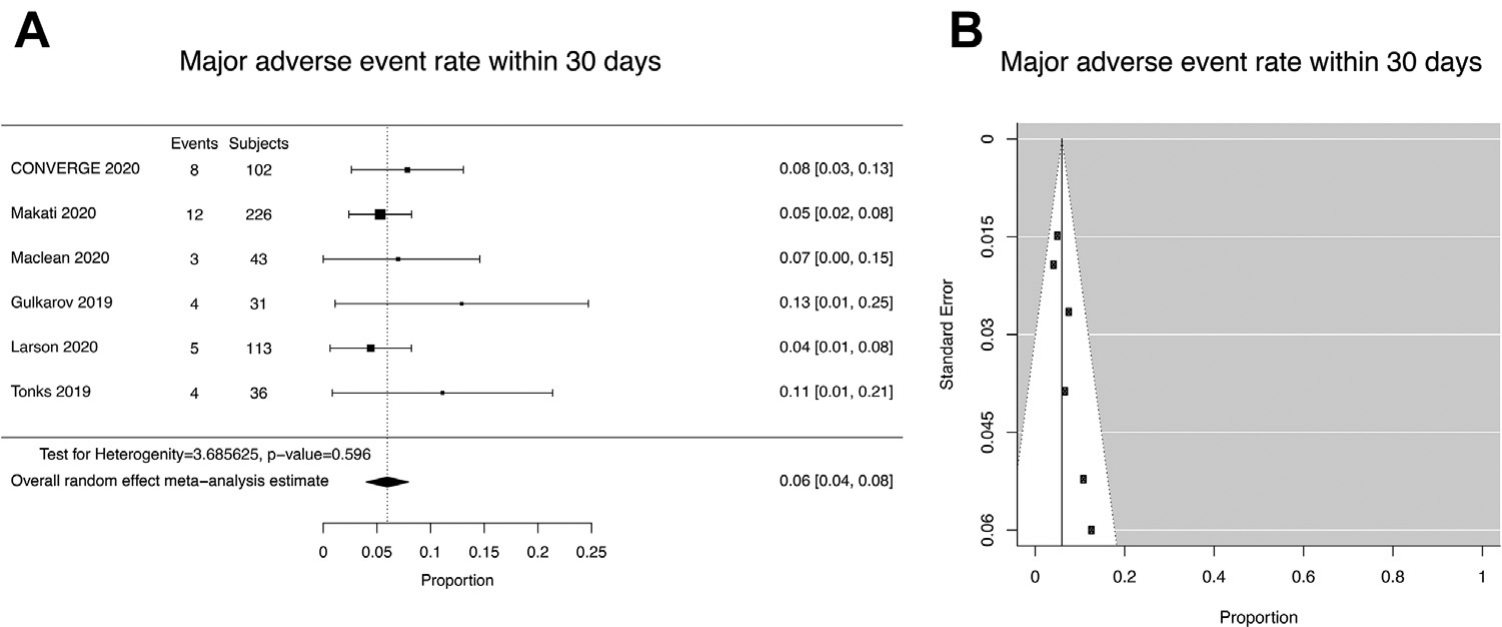
Meta-analysis of major adverse events (MAEs) within 30 days of Hybrid Convergent procedure. **A:** Forest plot of MAE rate within 30 days. **B:** Funnel plot of MAE rate within 30 days. MAEs were defined per CONVERGE protocol (2017 HRS consensus statement).

For the studies identified in this systematic review, a summary of MAEs that occurred within 30 days of the Hybrid Convergent procedure is shown in Supplemental Table 3. Five studies had MAE rates within 30 days ranging from 4% to 13%, in comparison with 8% with CONVERGE. Meta-analysis found the pooled 30-day MAE rate (random-effects model) to be 6% (95% CI: 4%–8% n = 551; Figure 3). No AEFs, tamponade from cardiac perforations, or periprocedural deaths occurred in these 6 studies. Heterogeneity was not significant (Q [df = 5] = 3.6856,*P* =.59550).

Three studies reported on AF burden. Owing to differences in how AF burden or burden reduction was reported, a meta-analysis of AF burden was not performed. A qualitative data summary is shown in Table 3. CONVERGE showed that a significantly greater proportion of patients treated with Hybrid Convergent procedure experienced ≥90% AF burden reduction at 12 and 18 months compared with patients treated with endocardial ablation alone. The studies by Larson and colleagues^20^ and Makati and colleagues^22^ showed 88%–95% of patients who had continuous monitoring during follow-up had AF burden ≤5% at 12 or more months post procedure.^20^^,^^22^

**Table 3:** Summary of atrial fibrillation burden analyses from identified studies

Study	AF burden	Time post procedure	Hybrid Convergent	Endocardial ablation	*P* value
De Lurgio et al 2020^10^	• ≥90% reduction in AF burden from baseline	• 12 months	80%	57%	*P* = .007
		• 18 months	74%	55%	*P* = .0395
Makati et al 2020^22^	• ≤5% AF burden	• 3–12 months†	94%	-	-
		• 12–24 months‡	88%	-	-
	• Residual AF burden	• 3–12 months†	1.1%	-	-
		• 12–24 months‡	8.5%	-	-
Larson et al 2020^20^	• ≤5% AF burden	• 12 months	94%	-	-
	• Mean residual AF burden	• 12 months	2.8%	-	-
		• 18 months	4.3%	-	-

AF = atrial fibrillation.

† Mean 7.3 months.

‡ Mean 19 months.

Two of the identified studies compared Hybrid Convergent procedure outcomes with endocardial RF catheter ablation outcomes. CONVERGE was a randomized controlled study of these 2 approaches, whereas Maclean and colleagues used propensity score–matched cohorts treated with endocardial catheter ablation or Hybrid Convergent procedures. These results are shown in Supplemental Table 4. Both individual studies reported statistically improved single-procedure clinical outcomes at 12 months with Hybrid Convergent procedures compared with endocardial catheter ablation, including off AADs.

## Discussion

### Efficacy and outcomes of hybrid ablation

Our systematic review identified 6 recent studies that described effectiveness and safety outcomes of the Hybrid Convergent procedure in 551 patients with primarily drug-refractory PersAF or LSPAF. In our meta-analysis, we found that among patients who had Hybrid Convergent ablation with contemporary methods, the rate of freedom from atrial arrhythmia was 69%, whereas the rate of freedom from atrial arrhythmias off AADs was 50%. While interpreting these data, it should be taken into consideration that the patients referred for convergent ablation are very high-risk patients for recurrence, with high body mass index, high prevalence of hypertension, and sleep apnea. Likewise, these patients have longer duration of AF—CONVERGE trial patients had persistent AF for 4.4 years on average, Maclean and colleagues had inclusion criteria of persistent AF more than 12 months, Gulkarov and colleagues had 48% with LSPAF, and Makati and colleagues had 60% of patients with LSPAF. If we compare this to other contemporary endocardial ablation studies on PersAF patients, STOP Persistent AF trial inclusion criteria was PersAF less than 6 months’ duration^23^ and the PRECEPT trial excluded patients with continuous AF for more than 12 months.^24^ The patients being referred for the Convergent procedure are generally patients deemed by the referring electrophysiologist likely to fail with a single endocardial ablation alone. The anticipated advanced substrate population unfortunately makes complete cessation of AADs more challenging and difficult. The CONVERGE trial compared the convergent ablation to endocardial ablation group; the rate of freedom from any atrial arrhythmias off AADs was 53.5% in the convergent group compared to 32% in the endocardial ablation–alone group. Likewise, the BELIEF trial reported a 12-month freedom from AF/AT off AADs after standard ablation as 28% and after standard ablation + empirical LAA isolation as 56%.^25^ At 18-month follow-up, STAR AF II reported freedom from AF off AADs as 38% and freedom from atrial arrhythmias off AADs as 32%.^26^ STOP persistent AF^23^ and CRYO4PERSISTENT AF^27^ had no clear data on the freedom from AF off AADs. These 2 trials only reported freedom from atrial arrhythmias on or off antiarrhythmic therapy. In this context, the Convergent ablation results are favorable.

Of the 6 studies that we evaluated, only Makati and colleagues^22^ reported outcomes data for PersAF and LSPAF populations separately and found that 85% of patients with PersAF and 70% of patients with LSPAF had freedom from AF/atrial flutter/AT on or off previously failed AAD. In PersAF and LSPAF, AF burden reduction and residual AF burden may be additional relevant endpoints that reflect an improvement in patient symptoms.^12^ CONVERGE showed a significantly greater proportion of patients who experienced ≥90% AF burden reduction at 12 and 18 months than with catheter ablation, and 2 other studies in the meta-analysis showed low residual AF burden through 12 months after Hybrid Convergent procedures. These clinical outcomes are favorable in the context of effectiveness rates published for endocardial catheter ablation alone.

Meta-analysis of MAEs that occurred within 30 days found a pooled rate of 6%. In the comparison studies included in the meta-analysis, the rate of MAEs was numerically higher than those undergoing endocardial catheter ablation alone.^10^^,^^21^ However, collectively across studies, individual MAEs occurred at rates in line with estimates for AF-related ablation.^12^ The most frequent event was pericardial effusion. These are typically delayed, inflammatory effusions (1–3 weeks after the procedure), likely in response to pericardiotomy and ablation, not cardiac perforation. In the 6 studies, 80% of these events were treated with pericardiocentesis or managed medically and 20% were treated with pericardial window.

Several of the studies discussed risk mitigation strategies that were implemented with experience with the procedure, including prophylactic use of anti-inflammatory drugs and postprocedure transthoracic and transesophageal echocardiograms to mitigate and monitor for delayed inflammatory pericardial effusions, respectively.^18^^,^^20^^,^^22^ Furthermore, 2 studies reported complication rates stratified by pericardial access type (transdiaphragmatic or subxiphoid). Both studies noted significantly decreased complication rates after transitioning to a subxiphoid approach to pericardial access. Larson and colleagues^20^ reported an overall complication rate of 3.8% with subxiphoid access compared with 23% with transdiaphragmatic access (*P* = .005). Similarly, Makati and colleagues^22^ reported that all periprocedural complications occurred with transdiaphragmatic access, whereas none occurred in cases where a subxiphoid approach was used (*P* = .012). A previous meta-analysis on convergent procedures that included 6 older studies using earlier lesion sets (ie, extracardiac maze or posterior wall box) and/or previous-generation unipolar RF devices found a pooled complication rate of 9.0% with a mortality rate of 1.7%, which was mostly attributed to AEFs.^28^ Our current review of recent studies found no AEFs and no periprocedural deaths. It should be noted that the reported population treated with these contemporary practices is still limited, given that the incidence of AEF is estimated to be approximately 0.02%–0.11% with traditional catheter ablation.^12^

There are several potential contributing factors to the observed improvement in these serious complications. There is a learning curve to the procedure that may have resulted in improved outcomes. The unipolar RF ablation catheter now has gone through various modifications, and the Visitrax and Numeris guided coagulation devices used in earlier studies have been replaced with EPi-Sense, the fourth-generation device, which has an added sensing function. EPi-Sense allows physicians to determine the type of tissue the device may be in contact with (atrial vs nonatrial). Marker indicators aid in visualization of the side of ablation energy delivery. Also, best practice recommendations are made to use an esophageal temperature monitor and saline irrigation during ablation to reduce potential for adjacent tissue heating and also to avoid ablating on the pericardial reflections. The procedure changed from an extracardiac maze approach with extensive epicardial ablation to a strategy focused on creation of linear parallel overlapping lesions on the posterior wall.

### Hybrid ablation vs catheter ablation

Current literature reports variable data on the outcomes of ablation therapy for AF. Outcome comparisons of endocardial, hybrid, and surgical ablation are confounded by differences in AF type, ablation strategy, and varying definitions of clinical endpoints. There are few data comparing the outcomes of catheter ablation vs hybrid ablation in the same study. Our review of contemporary studies found 2 studies that compared the outcomes of Hybrid Convergent ablation with endocardial catheter ablation alone: 1 randomized study^10^ and 1 propensity score–matched study.^21^ All of the patients in the study by Maclean and colleagues^21^ had LSPA^1^ and 42% of CONVERGE patients had LSPAF.^10^ Both studies found significantly improved effectiveness with Hybrid Convergent ablation compared with endocardial RF ablation on or off AADs. In CONVERGE (mean 53 months AF duration), primary effectiveness was 67.7% after Hybrid Convergent compared with 50.0% after catheter ablation.^10^ In Maclean and colleagues^21^ (mean 30–36 months AF duration), freedom from AF at 12 months was 60.5% on AADs or 37.2% off AADs after Hybrid Convergent compared with 25.6% on AADs and 13.9% off AADs after catheter ablation. Recent endocardial catheter ablation trials reported primary effectiveness of 54.8% through 12 months with cryoballoon ablation for PersAF (mean 7.2 months PersAF duration)^23^ and 61.7% through 15 months with endocardial catheter ablation for PersAF (mean 15.9 months PersAF duration, with 2 repeat ablations permitted during 6-month therapy consolidation period).^24^

### Quality assessment

This is a systematic review of 5 observational studies and 1 randomized study. Two out of the 6 studies had comparison groups, while the remaining 4 were single-arm studies. We took various measures to minimize the bias, starting with wide search criteria and using 2 comprehensive search engines to avoid appropriate studies being inadvertently left out. Using 2 well-utilized risk-of-bias assessment tools, the risk of bias of CONVERGE was determined to be low and the scores of the nonrandomized studies were within an acceptable range for cohort studies (6–8 points).

### Study limitations

Significant heterogeneity was detected in the effectiveness meta-analyses in this study, which may be in part owing to the relatively small number of total studies and patients included. Although most studies evaluated effectiveness at least 1 year post procedure, there were differences in the exact time point evaluated and also the method of rhythm monitoring. Endocardial ablation was performed exclusively with cryoballoon in 1 study, while 4 studies used exclusively RF endocardial ablation. Also the extent of endocardial ablation beyond PVI also varied among studies. However, this heterogeneity reflects the real-life practice of patient-tailored ablation approach, where the extent of ablation and lesion sets may be additionally based on induced arrhythmias during the procedure. Contemporary studies such as the PRECEPT trial^24^ also show such heterogeneity—the primary goal of the ablation procedure was PVI, although additional ablation targeting atrial substrate or non-PV triggers was permitted at operator’s discretion. One study included 10 patients who had LAA exclusion,^19^ which could have potentially influenced their clinical outcomes, given the benefit of LAA electrical isolation previously reported in the BELIEF trial.^25^ However, these patients represented less than 2% of the total in the meta-analysis. Female patients were under-represented. It has been shown that females may have different outcomes from endocardial ablation and more non-PVI triggers; therefore this could impact generalizability of the findings.^29^ Thus, it is recognized that these limitations and aforementioned aspects of heterogeneity, including the extent of endocardial ablation, rhythm monitoring type, number of patients, and history of prior ablation among the identified studies, should be considered carefully alongside the safety and effectiveness results from this meta-analysis.

## Conclusion

Because Hybrid Convergent ablation is a relatively new approach compared with surgical and catheter ablation, data on its use continue to emerge. Recent randomized controlled data from CONVERGE and other available published data support that it is an effective ablation strategy for PersAF and LSPAF. Patient outcomes were not reported separately for Hybrid Convergent ablation as a repeat ablation vs *de novo* procedure; therefore, a comparative meta-analysis was not possible. This is a relevant question for future evaluation. A study on Hybrid Convergent procedures performed with a previous-generation device did not find a significant difference in atrial arrhythmia or AF recurrence between patients who had prior ablation compared with those who did not have prior ablation.^30^ Five out of 6 studies in this analysis were performed in a single setting and 1 reported data from a staged setting; therefore, meta-analysis comparing outcomes from these approaches was not possible. Timing is often driven by institutional practices, but a formal comparison of outcomes has not yet been performed. Other future research could evaluate whether additions to the epicardial portion of the Hybrid Convergent procedure to target extrapulmonary vein arrhythmogenic regions potentially enhance clinical outcomes without adding significant risk, such as LAA exclusion or vein of Marshall ablation.
