# Supplementary material for: Hybrid Convergent ablation for atrial fibrillation: A systematic review and meta-analysis
Source: Heart Rhythm O2. 2022 May 16;3(4):396–404. doi: 10.1016/j.hroo.2022.05.006 (PMC9463711; doi:10.1016/j.hroo.2022.05.006)
Supplement: Supplementary Tables 1-4 [file mmc1.docx]

| **Supplementary Table 1. Newcastle-Ottawa Scale – risk of bias assessment for cohort studies** | | | | | |
| --- | --- | --- | --- | --- | --- |
|  | Makati et al. 2020 | Maclean et al. 2020 | Larson et al. 2020 | Tonks et al. 2019 | Gulkarov et al. 2019 |
| Selection  (max 4 points) | 3 | 4 | 3 | 3 | 3 |
| Comparability  (max 2 points) | 1 | 1 | 0 | 0 | 0 |
| Outcome  (max 3 points) | 3 | 3 | 3 | 3 | 3 |
| Total points  (max 9 points) | 7 | 8 | 6 | 6 | 6 |

**SUPPLEMENTAL MATERIAL**

| **Supplementary Table 2. Cochrane Risk of Bias tool for randomized trials (RoB 2) – risk of bias assessment for CONVERGE** | |
| --- | --- |
| Risk of bias domain | CONVERGE (DeLurgio et al. 2020) |
| Randomization process | Low |
| Assignment to intervention | Low |
| Missing outcome data | Low |
| Measurement of outcome | Low |
| Selection of the reported result | Low |
| Overall risk of bias | Low |

| **Supplementary Table 3** Effectiveness and safety outcomes of Hybrid Convergent ablation | | | | | | | |
| --- | --- | --- | --- | --- | --- | --- | --- |
| Study | Monitoring type | Monitoring frequency after blanking period | Rhythm evaluation timepoint | Freedom from atrial arrhythmias irrespective of AADs, % (n/N) | Freedom from atrial arrhythmias off AADs, % (n/N) | MAEs within 30 days^¶^ | MAE rate, % (n/N) |
| De Lurgio et al 2020^10†^ | ECG (100%), 24h Holter (100%)^‡^ | ECG at 3, 6, and 12 months;  24h Holter at 6 and 12 months | 1 year | 76.8 (76/99) | 53.5 (53/99) | 1 bleeding  1 bleeding with late pericardial effusion  3 pericardial effusions  1 stroke  1 phrenic nerve injury  1 TIA | 7.8 (8/102) |
| Makati et al 2020^22^ | ILR/ICD (53%), ECG (47%) | ECG, ILR, or device interrogation at 3, 6, 12, and 24 months | Mean 15.4 ± 6.5 months | 75 (151/201) | 53 (106/201) | 3 bleeding  6 pericardial effusions  2 phrenic nerve injuries  1 stroke | 5.3 (12/226) |
| Maclean et al 2020^21†^ | Pacemaker interrogation (plus Holter), Holter, ECG | ECG at 3 months,  72h Holter at 6 months,  ECG and symptom guided 72h Holter at 12 months | 1 year | 60.6 (26/43)^§^ | 37.2 (16/43) | 2 tamponade  1 phrenic nerve injury (palsy) | 7.0 (3/43) |
| Gulkarov et al 2019^18^ | ICD (74%), ECG and event monitors (26%) | ECG, event monitors, or ICD interrogation at 3, 6, 12 and then every 6 months | 1 year | 71 (22/31) | NR | 2 pericardial effusions with cardiac tamponade  2 stroke | 12.9 (4/31) |
| Larson et al 2020^20^ | ILR (69%), CIED (12%), event monitors (19%) | Event monitors at 6 and 12 months or as clinically indicated for patients without CIEDs/ILRs;  ECG from in-hospital and office visits also reviewed for all patients | 1 year | 53 (60/113) | NR | 3 pericardial effusions with tamponade  2 excessive bleeding | 4.4 (5/113) |
| Tonks et al 2019^19^ | Holter, ILR, CIED, ECG | ECG, ILR, Holter, or device interrogation at 3, 6, and 12 months | 1 year | 78 (28/36) | NR | 1 phrenic nerve injury (palsy)  1 cardiac tamponade  2 pericardial effusions requiring pericardiocentesis | 11.1 (4/36) |
| ^†^Single procedure success rates reported, ^‡^7-day Holter performed at 18 months but data are not included in meta-analysis; ^§^Freedom from atrial fibrillation was reported at 12 months. ^¶^Major adverse events defined as: cardiac tamponade/perforation, severe pulmonary vein stenosis, excessive bleeding, myocardial infarction, stroke, transient ischemic attack (TIA), atrioesophageal fistula, phrenic nerve injury, and death.  AAD = anti-arrhythmic drug; CIED = cardiac implantable electronic device; ECG = electrocardiography; ICD = implantable cardioverter defibrillator; ILR = implantable loop recorder; MAE = major adverse event; NR = not reported. | | | | | | | |

| **Supplementary Table 4** Summary of studies comparing hybrid convergent procedure and catheter ablation | | |
| --- | --- | --- |
| Freedom from atrial arrhythmias irrespective of AADs | | |
|  | Hybrid convergent | Catheter ablation |
| CONVERGE | 76.8% (77/99) | 60.0% (30/50) |
| Maclean et al. 2020 | 60.5% (26/43) | 25.6% (11/43) |
|  | | |
| Freedom from atrial arrhythmias off AADs | | |
|  | Hybrid convergent | Catheter ablation |
| CONVERGE | 53.5% (53/99) | 32.0% (16/50) |
| Maclean et al. 2020 | 37.2% (16/43) | 13.9% (6/43) |
